# Corrosion Inhibition Mechanism and Stability of Quinic Acid as a Green Corrosion Inhibitor on Mild Steel

**DOI:** 10.1002/open.202500322

**Published:** 2026-01-15

**Authors:** Serap Toprak Döşlü, Leyla Ercan

**Affiliations:** ^1^ Department of Nutrition and Dietetics Faculty of Health Sciences Mardin Artuklu University 47100 Mardin Turkey; ^2^ Department of Health Culture and Sports Mardin Artuklu University 47000 Mardin Turkey

**Keywords:** corrosion inhibition, density functional theory, green inhibition, mild steel, quinic acid

## Abstract

Although corrosion prevention methods have been studied for many years, they still maintain their relevance and popularity. Today's metal protection methods are desired to be cheap, easy to use, permanent, and effective, as well as environmentally friendly. Organic‐based inhibitors are preferred due to their effectiveness and environmental benefits. Among these, organic acids, such as quinic acid, are particularly valued for their corrosion inhibition properties. Quinic acid, an organic acid found in various plants, serves as an effective corrosion inhibitor for mild steel in 0.5 M HCl solutions. This study evaluates its corrosion inhibition efficiency and stability under different storage conditions. Electrochemical techniques, including electrochemical impedance spectroscopy and polarization curve analysis, are employed to assess the inhibition performance. Surface characterization is conducted using scanning electron microscopy, atomic force microscopy, energy‐dispersive X‐ray spectroscopy, and contact angle measurements. Additionally, density functional theory analysis is performed to elucidate the molecular interactions of quinic acid. Experimental results demonstrate that quinic acid, at a concentration of 80 ppm in 0.5 M HCl, achieves a corrosion inhibition efficiency of 92% and maintains stability for up to 144 h. Environmentally friendly quinic acid has a high potential for use as inhibitors of mild steel corrosion.

## Graphical abstract

It has been found that quinic acid, an organic acid found in various plants, exhibits a high level of corrosion inhibition activity in preventing HCl‐induced corrosion and maintains its stability up to 144 h. Environmentally friendly quinic acid has a high potential for use as corrosion inhibitors in mild steel.

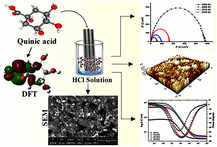

## 1 Introduction

Mild steel (MS) is widely utilized as a building material in many areas of the industry because of its low cost and mechanical properties.^[^ ^1^ ^]^ Corrosion is a major problem for many products in which metal alloys are used, such as in the iron and steel industries.^[^ ^2^, ^3^ ^–^ ^4^ ^]^ Strong acid solutions such as HCl and H_2_SO_4_, which are widely used in industry, lead to the corrosion of metal and metal alloys.^[^ ^4^ ^,^ ^5^ ^]^ Therefore, new inhibitors are sought to reduce or eliminate the corrosion process.^[^ ^6^ ^,^ ^7^ ^]^

Studies to eliminate the damage caused by corrosion and to find natural and cheap corrosion protectors are of great interest.^[^ ^8^ ^]^ In addition, due to their economic and environmental friendliness, the utilization of green corrosion inhibitors in the fields of chemistry and chemical engineering is especially important in the application areas such as organic coating, alloy, and the synthesis of porous materials in the industry.^[^ ^9^ ^]^ These studies also aim to elucidate the stability of natural components and the mechanisms of inhibition upon corrosion. Thus, it is desirable to discover inhibitors with effective anticorrosion effects. For this purpose, research has been carried out on the ability of different parts of various plants and fruits to prevent the corrosion effect on metal alloys.^[^ ^9^, ^10^ ^–^ ^11^ ^]^ These studies utilize both crude plant extracts and specific bioactive compounds isolated or synthesized from plants to evaluate their corrosion inhibition properties. The environmental toxicity of chemical inhibitors such as pyridines, hydrazines, and azoles encourages the search for environmentally friendly compounds in this area.^[^ ^12^ ^–^ ^14^ ^]^ Consequently, organic compounds, particularly those derived from natural sources, are increasingly employed as corrosion inhibitors to protect metals in aggressive solutions.^[^ ^13^ ^,^ ^15^ ^,^ ^16^ ^]^

Organic acids are compounds that give flavor to various fruits and plants.^[^ ^17^ ^]^ Organic acids in fresh herbs mostly include citric acid, tartaric acid, malic acid, and quinic acid.^[^ ^17^, ^18^ ^–^ ^19^ ^]^ Quinic acid, one of these organic acids, is a cyclohexane carboxylic acid predominantly derived from plants such as cinchona bark, coffee beans, sweet potatoes, apples, and peaches.^[^ ^20^ ^]^ There are studies on the health and pharmacological properties of quinic acid in the literature.^[^ ^21^, ^22^ ^–^ ^23^ ^]^ Some of these are studies that reveal properties such as antimicrobial activity,^[^ ^24^, ^25^ ^–^ ^26^ ^]^ antidiabetic, anti‐inflammatory,^[^ ^27^ ^]^ DNA repair,^[^ ^28^ ^]^ inhibition effect on NF–Kb,^[^ ^29^ ^]^ and anticarcinogenic.^[^ ^23^ ^,^ ^30^ ^]^ However, there is no study investigating the impact of quinic acid on metals and alloys used in industry. Quinic acid may have the potential to be effective in preventing corrosion by creating a hydrophilic layer in an acidic solution, thanks to the OH groups in its structure. In this study, the impact of quinic acid on corrosion caused by HCl and its resistance to this corrosion were investigated. Additionally, theoretical calculations of quinic acid were examined by density functional theory (DFT) analysis. The corrosion resistance of the green inhibitor quinic acid was investigated using electrochemical methods. Rather than any mixture or extract, the effect of this compound on corrosion has been directly revealed, thus facilitating applications to prevent corrosion caused by HCl and other strong acids.

## 2 Results and Discussion

**Figure 1:**
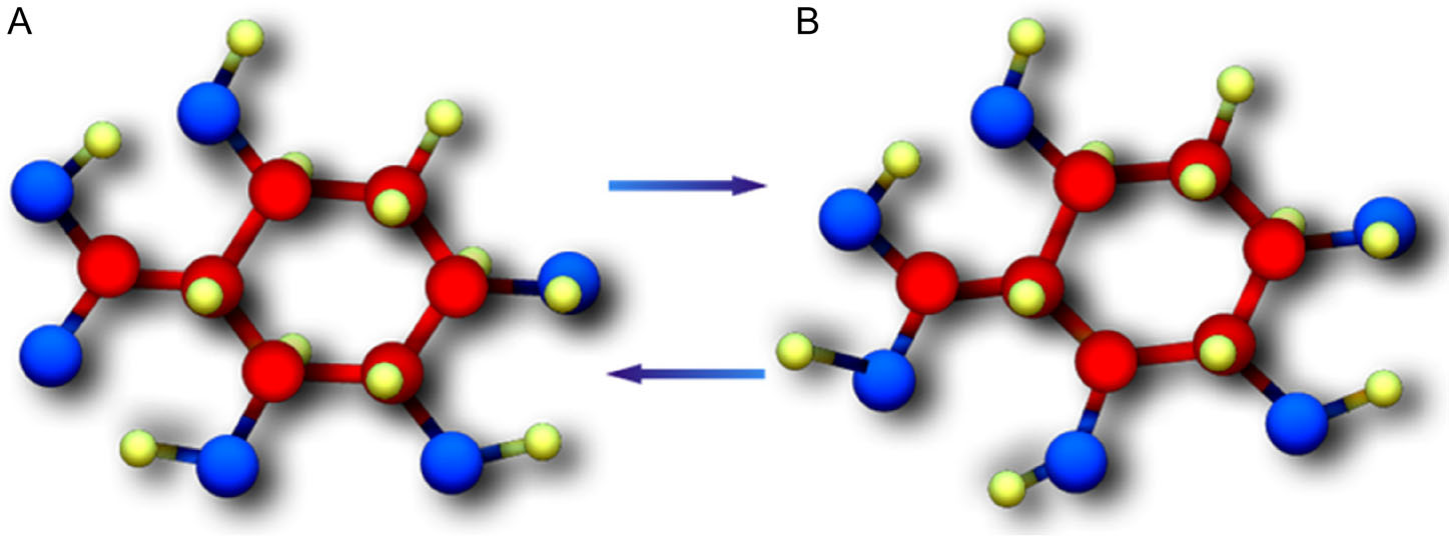
A) Neutral and B) protonated forms of quinic acid.

**Figure** **1** shows the neutral and protonated states of quinic acid. Quinic acid is an essential natural acid. Numerous quinic acid derivatives exhibiting diverse biological activity have been isolated from natural sources. Quinic acid is found in numerous species such as coffee beans, tobacco leaves, apples, carrot leaves, pears, peaches, plums, vegetables, and so on.^[^ ^31^ ^,^ ^32^ ^]^ Many plants containing quinic acid have been used as inhibitors against corrosion due to their many advantages, such as high inhibition efficiency, worldwide availability, environmental friendliness, and easy production.^[^ ^33^, ^34^ ^–^ ^35^ ^]^

The red balls represent C atoms, the blue balls represent O atoms, and the yellow balls represent H atoms.

**Figure 2:**
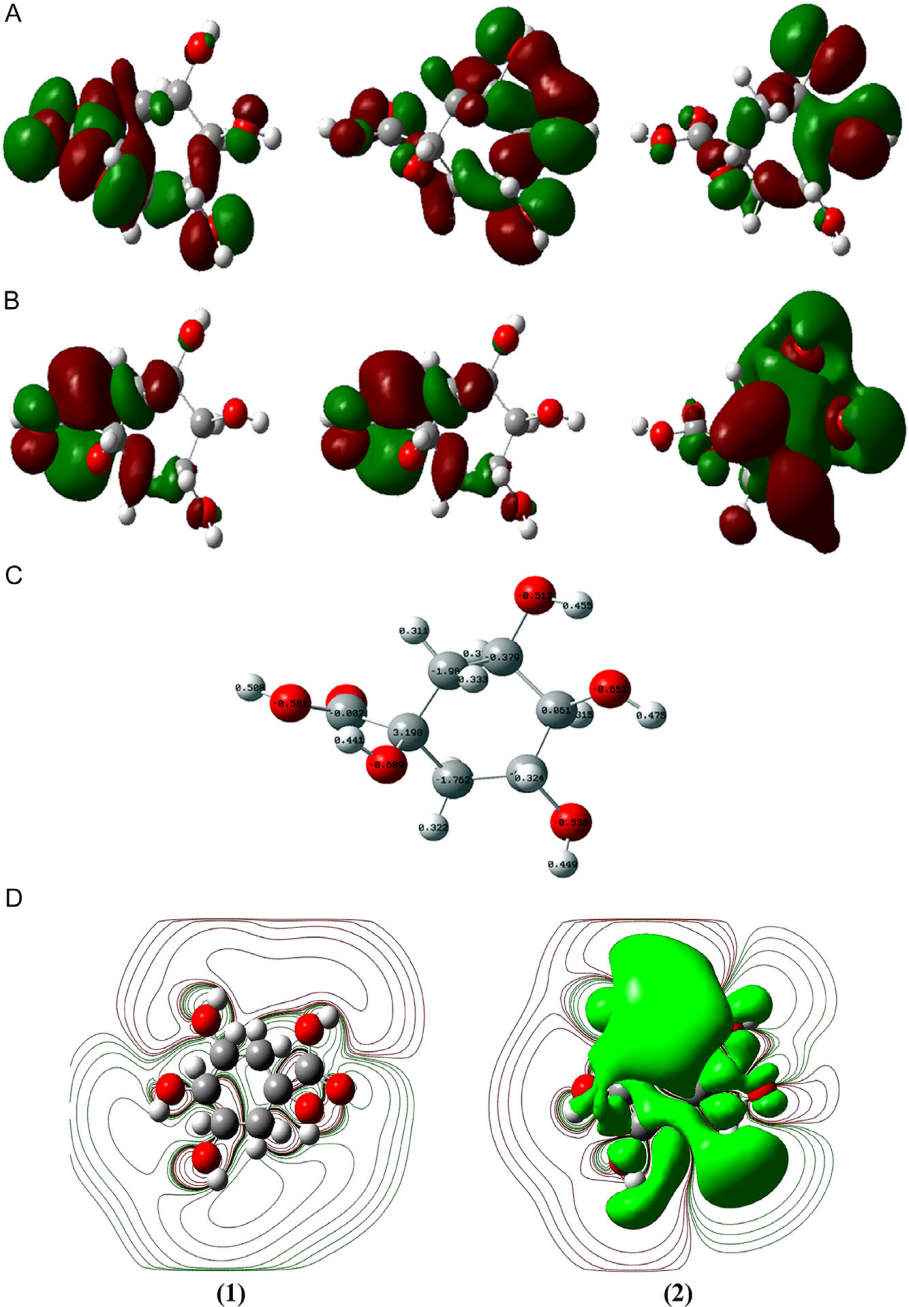
A) (Homo), B) (Lumo) orbitals of quinic acid, C) Mulliken charges, D1) LUMO ESP, and D2) HOMO ESP.

The atoms of the molecule, their charges, orbitals, and their interactions in an acidic solution are important in the anticorrosion effect of quinic acid. **Figure** **2** displays the highest occupied molecular orbital (HOMO), lowest unoccupied molecular orbital (LUMO) orbitals, and Mulliken atomic charges of quinic acid. HOMO (A), LUMO (B) orbitals, Mullikene charges (C), and LUMO Electrostatic Potential (ESP) (D1), HOMO ESP(D2) of quinic acid are displayed in Figure 2. The ESP is explanatory for electrophilic nature, nucleophilic reactions, and hydrogen bonding interactions. On molecular ESP surfaces, the most negative regions are shown in red and define electrophilic reactions, whereas blue and green colors represent the most positive regions and nucleophilically dominant regions.^[^ ^36^ ^]^

The sum of Mulliken charges is zero. Calculation of quinic acid in water as solvent was performed using the RB3LYP method and 3‐21+G basis set. Calculations were made by utilizing the Gaussian 09 package. 51 *α* and 51β electrons, the nuclear repulsion energy was determined as 912.702 Hartrees. The error in the total polarization charges was = 0.0165. The HOMO and LUMO values were discovered to be −9.324 and −3.337 eV, respectively. The results demonstrated that quinic acid is a suitable anticorrosion agent candidate for MS in an HCl environment. A molecule is a Lewis acid if its electronegativity (ꭗ) is higher and a Lewis base if it is lower.^[^ ^37^ ^]^ DFT calculations of quinic acid are reported in **Table** **1**. The dipole moment was 3.81 Debye.

**Table 1:** DFT calculations of quinic acid.

Compound	E_HOMO_	E_LUMO_	Bandgap (ΔE)	µ	ɳ	s	ꭗ	*ω*
Quinic acid	−9.324	−3.337	5.986	−0.039	2.993	1.496	6.330	0.002

In the calculations given in Table 1, the bandgap was found to be 5.986 eV, E_LUMO_: −3.337 eV, E_HOMO_: −9.324 eV. Chemical potential (µ), electrophilicity index (*ω*), chemical softness (s), ꭗ, and chemical hardness (ɳ) values, which are important in evaluating the corrosion inhibitory effect of quinic acid, were also determined. Among the DFT‐derived descriptors, *χ* and μ are closely associated with the acidity of the solution, as they reflect the electron‐accepting ability of quinic acid in the acidic medium. A lower chemical potential and higher electronegativity generally indicate stronger acid character in molecular systems.

**Figure 3:**
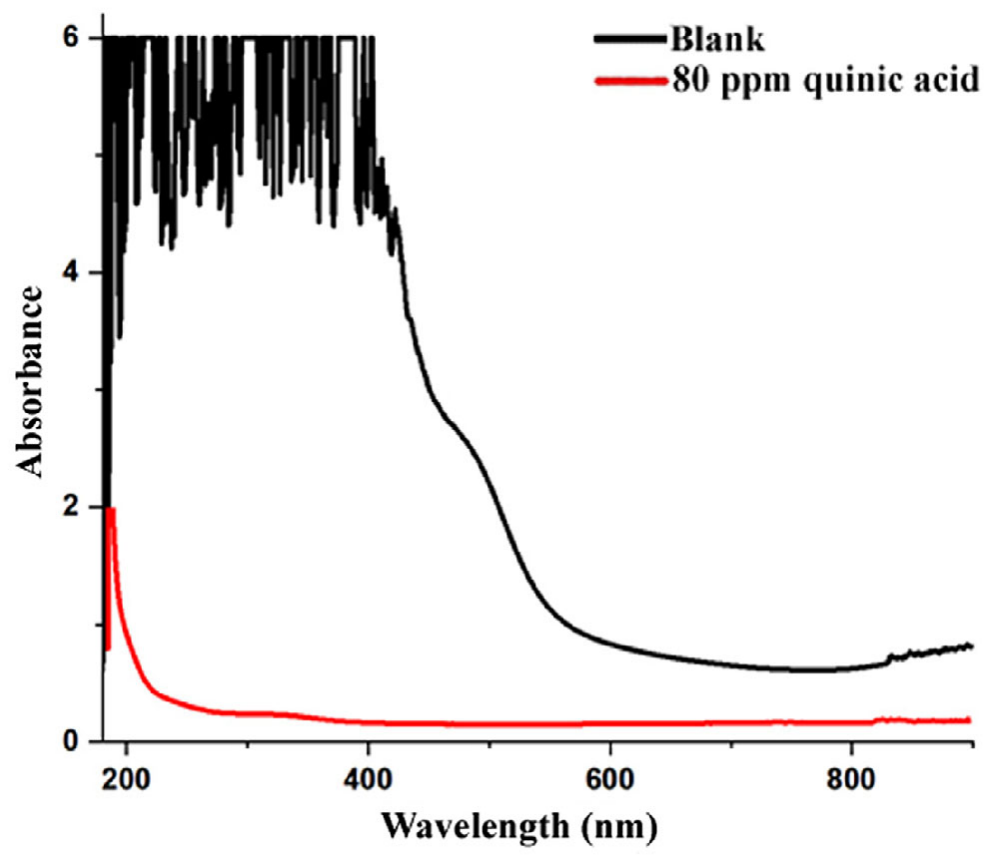
UV–VIS results of blank and 80 ppm quinic acid solutions (144 h after MS immersion).

To determine the corrosion inhibition effect of quinic acid, MS was submerged in blank (0.5 M HCl) and 80 ppm quinic acid (quinic acid + 0.5 M HCl) solutions for 144 h, and the absorbances of the solutions were measured. The measurement results in the UV–VIS spectrophotometer are given in **Figure** **3**.

The corrosion‐inhibitory impact of quinic acid on MS is illustrated in the absorbance measurements given in Figure 3. After 144 h of immersion of steel discs in 0.5 M HCl solution and quinic acid solution prepared in 0.5 M HCl, it was noted that the quinic acid solution was much lighter in color when both solutions were compared. Because the HCl solution without the inhibitor caused more corrosion, the color of this solution was darker than that with the inhibitor. The increased absorbance observed in the blank solution is likely due to the presence of dissolved iron ions (primarily Fe^2^^+^ and Fe^3^^+^) released from the MS surface in the absence of an inhibitor. In contrast, quinic acid may interact with Fe^2^^+^ions through chelation, forming complexes that are either retained in solution or adsorbed onto the steel surface. This adsorption may contribute to the formation of a protective film, thereby reducing the rate of corrosion and resulting in lower absorbance values in the quinic acid‐treated solution. This result indicates the inhibiting impact of this compound on corrosion.^[^ ^13^ ^]^

**Figure 4:**
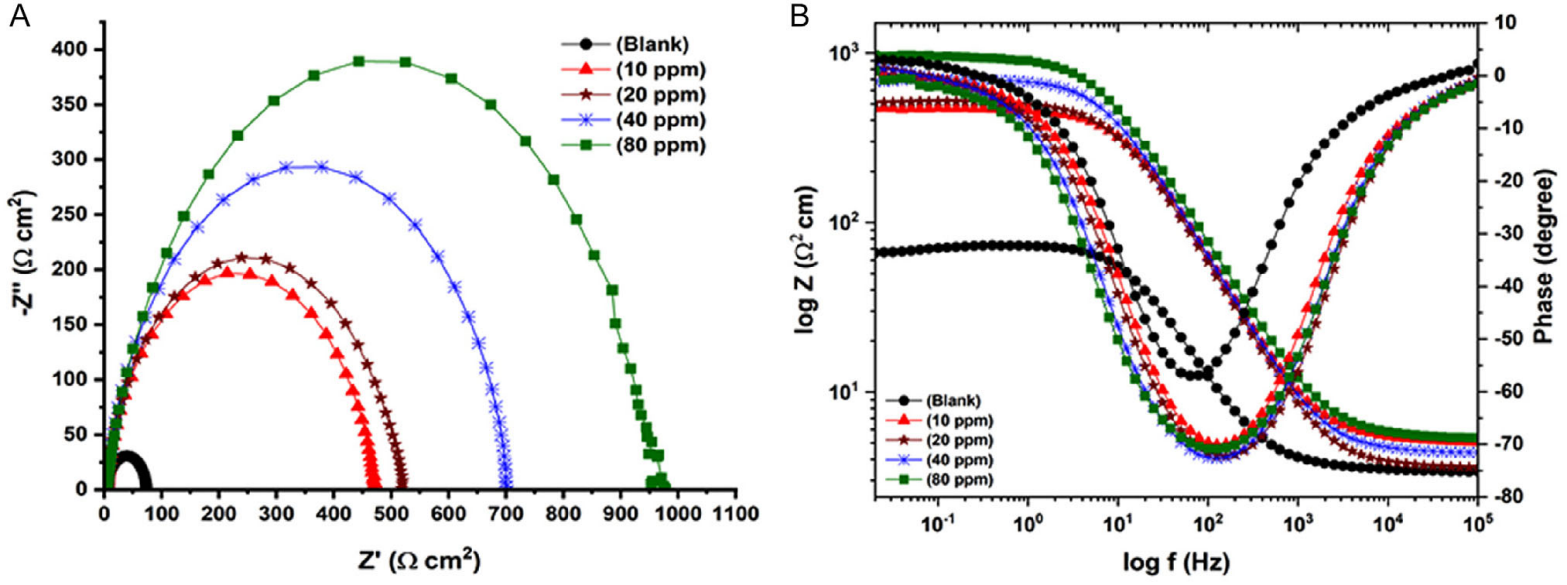
A)Nyquist B) and Bode curves of MS immersed for 1 h in 0.5 M HCl solution containing blank (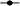), 10 (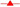), 20 (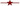), 40 (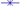), and 80 (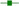) ppm quinic acid.

The ability of quinic acid to reduce corrosion on MS was analyzed utilizing electrochemical impedance spectroscopy (EIS) at open circuit potential (OCP) after 1 h immersion and under distinct experimental conditions. **Figure** **4** displays the impedance spectra in both Nyquist and Bode plots in solution without inhibitor and solution with inhibitor at varying concentrations. Every impedance spectrum in the Nyquist plots in Figure 4a consists of a suppressed semicircle with a diameter that rises with a rising concentration of quinic acid in HCl solution. This result verifies the corrosion inhibition effect of quinic acid and the efficient adsorption of its inhibitory property on the MS surface.^[^ ^38^ ^,^ ^39^ ^]^ The Bode plots are shown in Figure 4b. The phase angle is also altered toward high frequency. These indicators are unambiguous proof of the adsorption of quinic acid molecules on the surface of the working electrode.^[^ ^35^ ^]^

**Figure 5:**
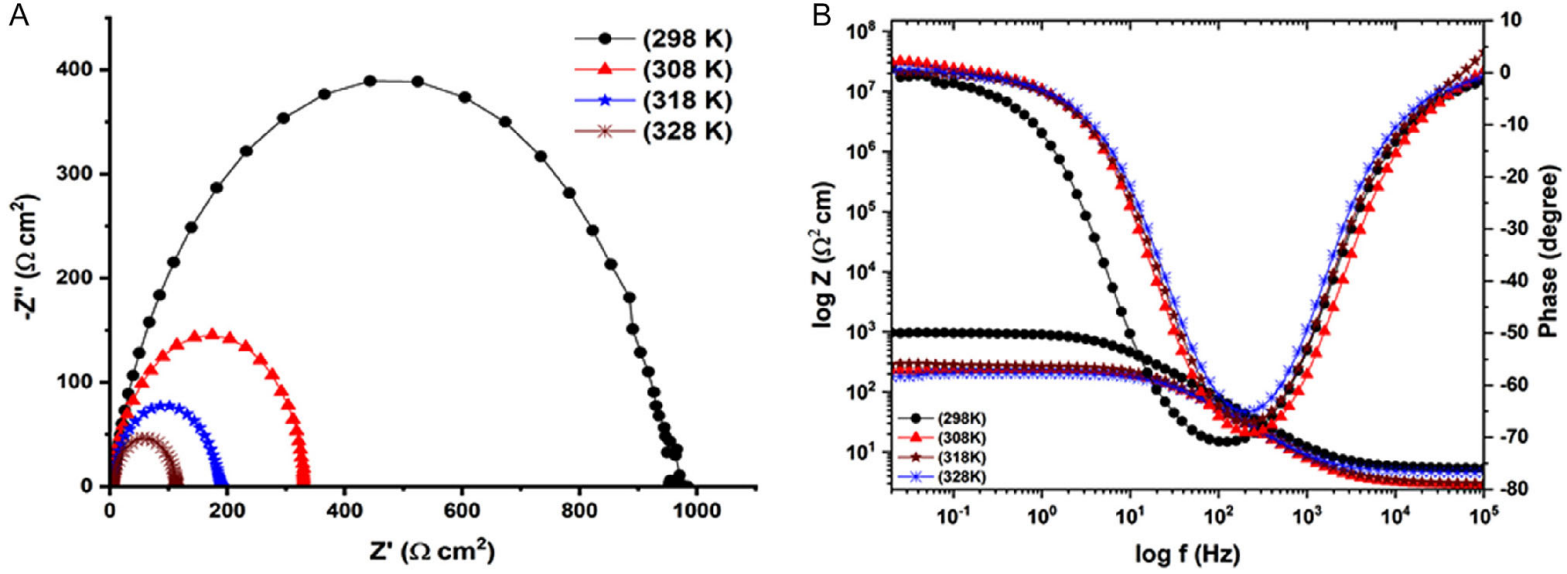
A) Nyquist B) and Bode curves of MS immersed in 0.5 M HCl solution containing 80 ppm quinic acid for 1 h at 298 (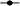), 308 (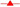), 318 (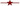), and 328 (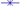) K.

Nyquist and Bode curves of MS (with an inhibitor and without an inhibitor) are displayed in **Figure** **5** (at distinct temperatures). The shape of the Nyquist plot for each EIS spectrum seems to have a semicircle, which may represent the formation of an inhibitor film. The semicircle is severely the same at all temperatures. These could result from the tightly packed inhibitor film formed on the metal surface. Therefore, even at high temperatures, quinic acid can protect MS against corrosion.

**Figure 6:**
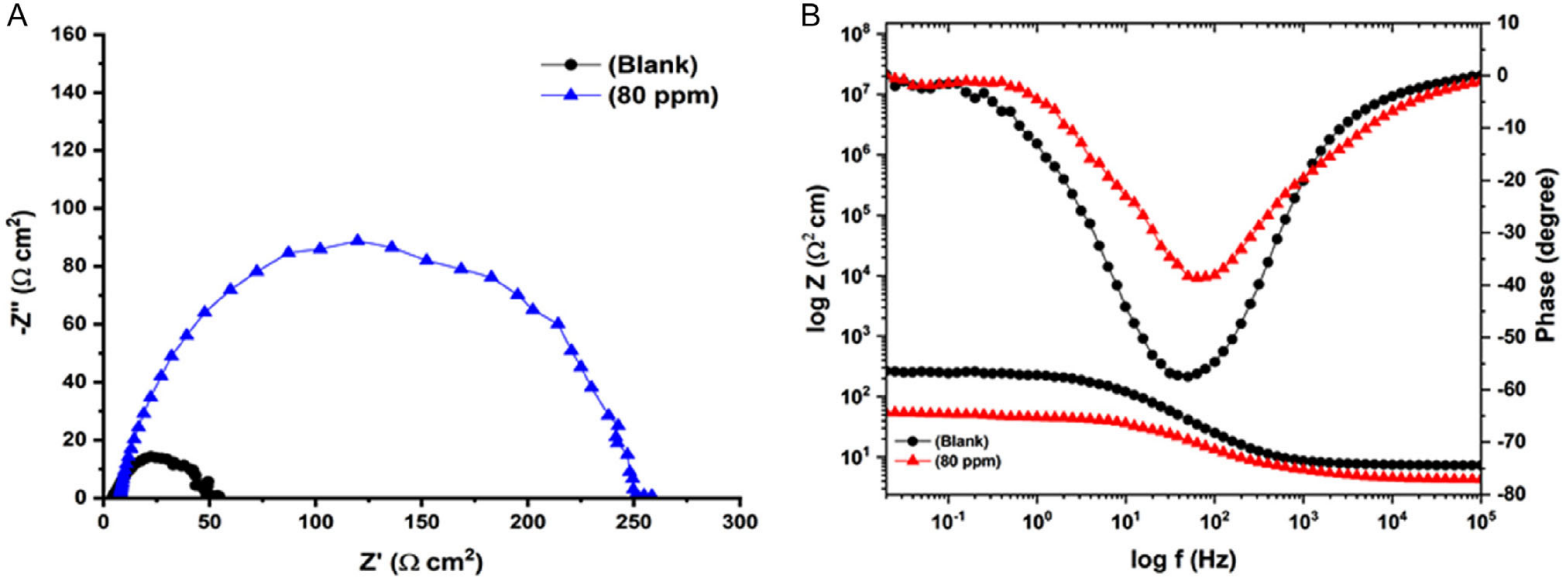
A) Nyquist B) and Bode curves of mild steel immersed in 0.5 M HCl solution containing blank (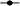) and 80 (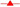) ppm quinic acid for 144 h.

Nyquist and Bode curves of MS (with an inhibitor and without an inhibitor) are shown in **Figure** **6** (for 144  h). The effect of the immersion time on the inhibition efficiency of quinic acid was investigated by EIS methods. The Nyquist and Bode plots of the steel in 0.5 M HCl solution without and with 80 ppm quinic acid were given in Figure 6a, b at different exposure times, respectively. One suppressed capacitive loop was observed at the Nyquist plots of the steel for uninhibited and inhibited corrosive solutions. With the exception of the appearance of Nyquist plots of the MS, the diameters of these capacitive loops significantly changed with exposure time. Especially, the steel electrode immersed in the blank solution was strongly affected by elevated time.

**Figure 7:**
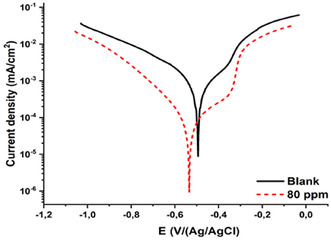
Polarization curve of mild steel submerged for 144 h in 0.5 M HCl solution containing blank (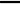) and 80 (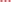) ppm quinic acid.

In quinic acid's presence, cathodic and anodic current density values dropped as shown in **Figure** **7**. In quinic acid's presence, the corrosion potential values were almost identical, affecting both cathodic and anodic reactions; therefore, quinic acid can be described as a mixed‐type inhibitor.^[^ ^40^, ^41^ ^–^ ^42^ ^]^

**Figure 8:**
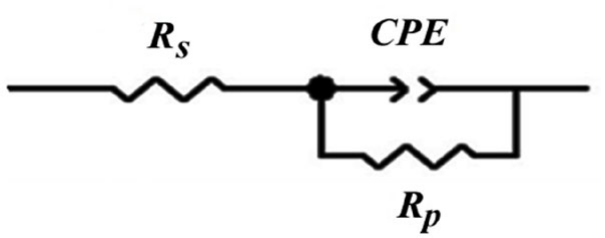
Equivalent circuit obtained from EIS data (*R*s: solution resistance, *R*p: polarization resistance, CPE: double layer capacitance).

The polarization resistance of MS in 0.5 M HCl solution was found to be 38.5 ohm cm^−2^. When we added 10, 20, 40, and 80 ppm quinic acid to 0.5 M HCl solution, the polarization resistances were measured as 234, 257, 350, and 480 ohm cm^−2^, respectively. Equivalent circuit and electrochemical parameters are presented in **Figure** **8** and Table **2**.

**Table 2:** Fitting results of EIS data and electrochemical parameters derived from EIS measurements for MS obtained 0.5 M HCl solution in the absence and presence of various concentrations of quinic acid at 298 K.

*C*_inh_ [ppm]a)	*CPE* *Y* ^*0*^[10 ^−4^ s^−1^ ^n^ Ω^−1^ cm^−^^2^]	n	*R*p[Ω cm^2^]	*R*s[Ω cm^2^]	*η*%
0 + 0.5 M HCl	56	0.90	38.5	1.5	–
10 ppm QA + 0.5 M HCl	58	0.90	234	2.8	83.5
20 ppm QA + 0.5M HCl	60	0.88	257	1.8	85.0
40 ppm QA + 0.5 M HCl	36	0.90	350	2.2	89.0
80 ppm QA + 0.5 M HCl	58	0.86	480	2.5	92.0

a) QA: Quinic acid, Rs: solution resistance, Y^0^: proportionality coefficient, Rp: polarization resistance, CPE: double layer capacitance, n: phase shift.

Based on the impedance plots, the electrical equivalent circuit diagrams exposed in Figure 8 were modeled for the MS/solution interface in the absence and presence of quinic acid.

In these diagrams, *R*s expresses the solution resistance and *R*p represents the polarization resistance at the metal/solution interface. CPE denotes the constant phase element and *n* is the phase shift, which can be described as a measure of the surface irregularity. The polarization resistance, Rp, for the uninhibited and inhibited solutions. Essentially, in an actual system, the capacitive loop deviates from the ideal semicircle, resulting in *n *≠ 1. The associated corrosion inhibition performance of the MS in the absence and presence of quinic acid at various concentrations is outlined in Table 1. As evident from the table, the effectiveness accrues as the concentration increases. Adsorbed at the MS/solution interface, quinic acid effectively reduces the active surface area of MS. This reduction may be associated with corrosion protection. MS exhibited supreme resistance due to the inhibitory effect of 80 ppm quinic acid solution.

**Figure 9:**
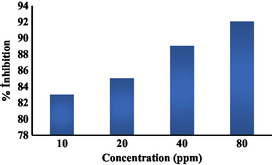
Graph of % corrosion inhibition on the steel of quinic acid solutions at different concentrations.

**Figure 10:**
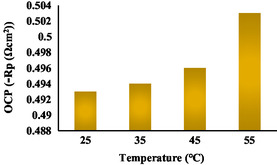
Open circuit potential change graph of steel submerged in 0.5 M HCl solution containing 80 ppm quinic acid solutions at different temperatures.

The corrosion inhibition graph at distinct concentrations and the graphs showing the change in OCP with temperature can be seen in Figure **9** and **10**. These graphs show that the increase shows the stability of quinic acid in corrosion inhibition.

**Figure 11:**
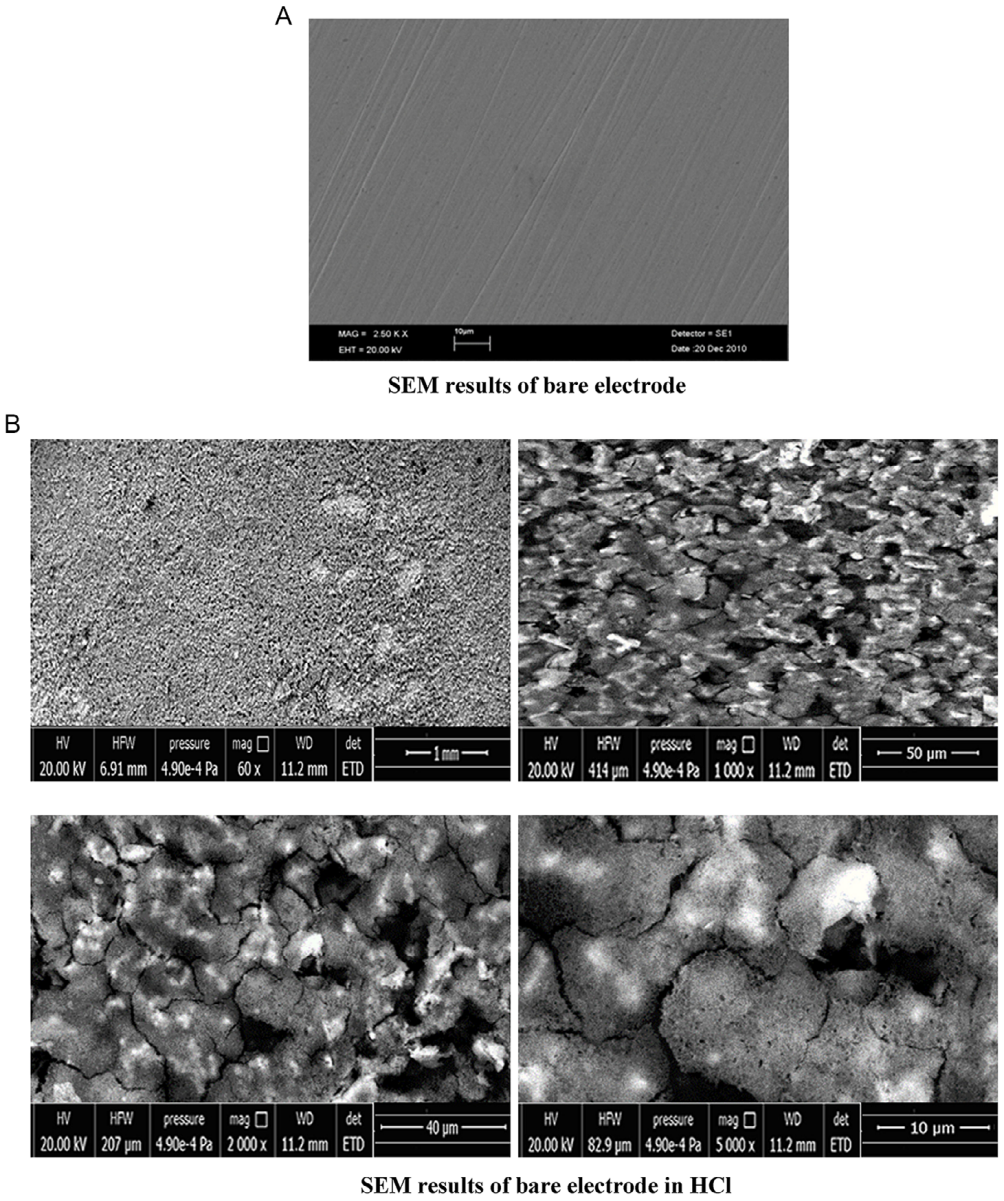
A) SEM results of bare electrode. B) SEM image of the electrode in HCl solution without inhibitor.

**Figure** **11** shows the change on the MS electrode surface. The scanning electron microscopy (SEM) image (Figure 11B) reveals widespread surface degradation characterized by irregular, noncrystalline deposits, likely indicating generalized corrosion with the formation of amorphous iron oxides. This morphology suggests that in the absence of an inhibitor, aggressive HCl attack leads to uncontrolled surface oxidation and pitting.

**Figure 12:**
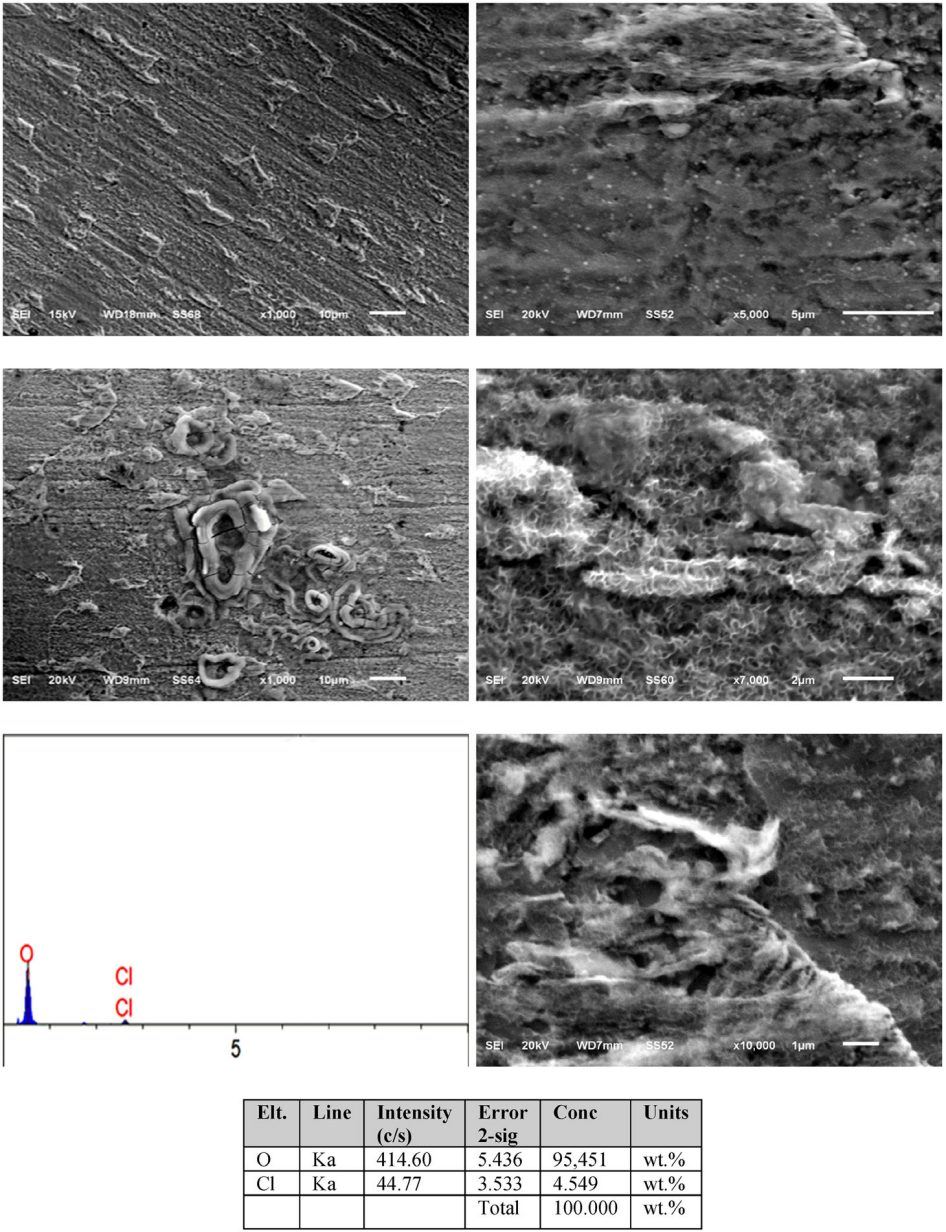
SEM and EDX results of MS submerged in a solution containing 0.5 M HCl containing 80 ppm quinic acid solution for 144 h.

**Figure** **12** shows SEM and energy‐dispersive X‐ray spectroscopy (EDX) results of MS after 144 h in 0.5 M HCl + 80 ppm quinic acid solution. The images illustrate localized surface features. The presence of O indicates possible oxide film formation. The SEM images in Figure 12 clearly show that the inhibitor can protect the MS surface from corrosion (with a reduction in corrosion).

**Figure 13:**
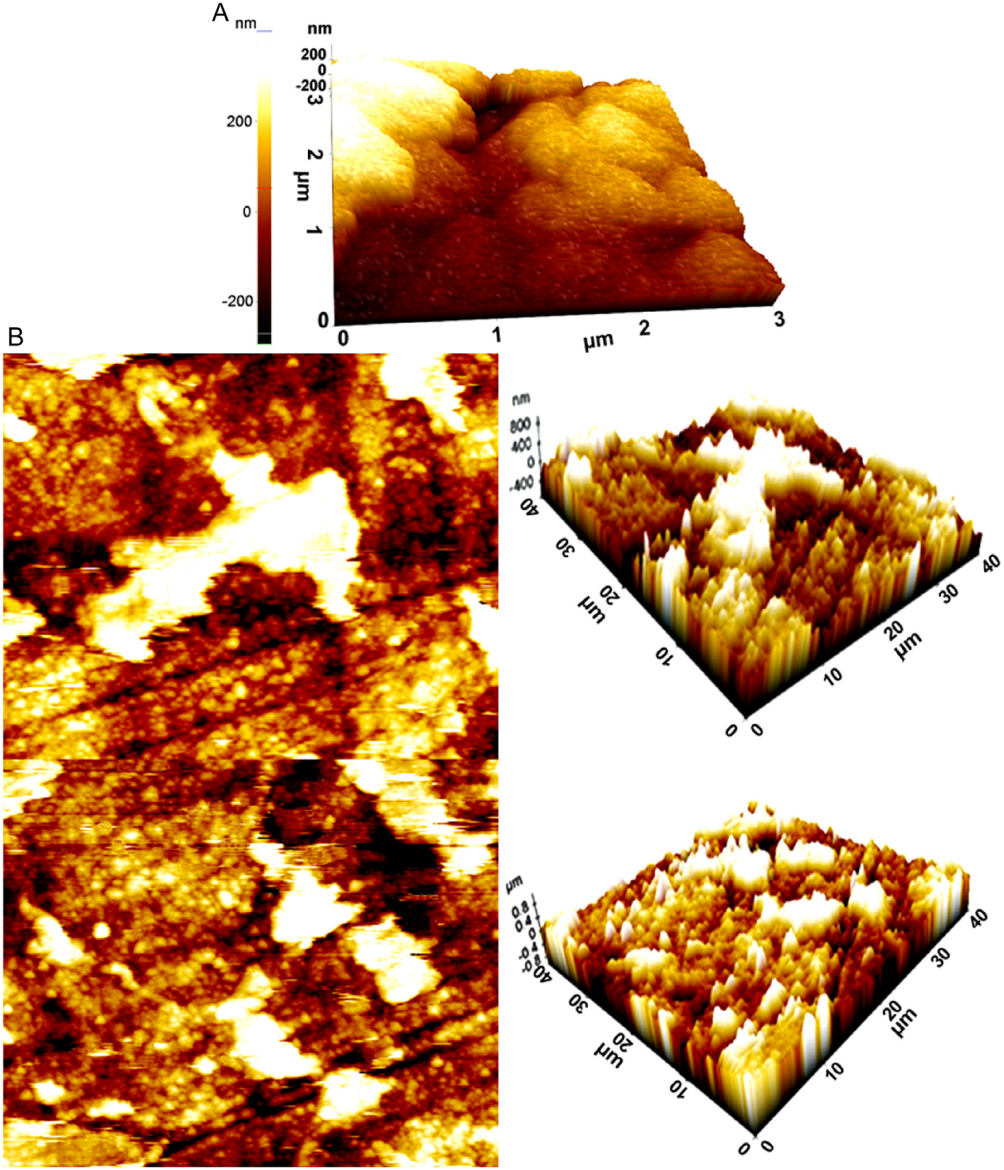
A) AFM results of electrode in HCl solution without inhibitor for 144 h, B) AFM and topography results of MS submerged in 0.5 M HCL solution containing 80 ppm quinic acid solution for 144 h.

The effectiveness of quinic acid in preventing corrosion is also shown by the atomic force microscope (AFM) and Topography results in **Figure** **13**. According to AFM and Topography results cleavages and corrosion are more on the surface of the noninhibitor electrode. The observed surface roughness variations support the proposed inhibition mechanism through surface adsorption and morphology alteration. These images may signify the presence of iron‐adsorbed quinic acid complexes and crystalline iron oxides that hinder corrosion on the electrode surface. These complexes form a protective layer on the metal surface, slowing down both anode and cathode reactions and considerably reducing the corrosion rate.^[^ ^43^ ^]^

**Figure 14:**
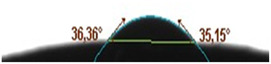
Contact angle of MS submerged in 0.5 M HCl solution comprising 80 ppm quinic acid for 144 h.

The hydrophilic or hydrophobic features of the surface give important clues about the arrangement of molecules adsorbed on the surface and the interplay of corrosive compounds with the surface or their passage through the film. One of the ways to reach this conclusion is to assess the contact angles.^[^ ^4^ ^,^ ^44^ ^]^ Contact angle views of the MS surface after 144 h exposure to 0.5 M HCl solution added 80 ppm quinic acid for 144 h are shown in **Figure** **14**.

The average contact angle of MS immersed in the test environment for 144 h is 35.76°. This value is less than 90° and indicates that the film is hydrophilic. The MS's average contact angle showed that the molecule adsorbed by the steel surface created a hydrophilic coating.

**Figure 15:**
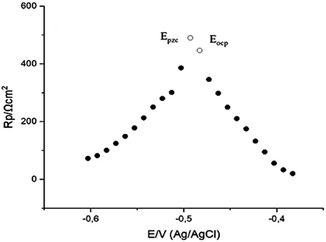
*E*_opc_ and *E*_pzc_ curves of an 80 ppm solution of quinic acid on MS.

The adsorption of organic substances on metal can be chemical, physical, or both chemical and physical. While physical adsorption is the electrostatic interaction between the metal surface and the inhibitor in solution, chemical adsorption can be expressed via charge division or charge transfer from the inhibitor to the metal surface. The electrical field at the solution/metal interface resulting from inhibitor adsorption determines the surface charge of the electrode. This surface charge is calculated by the following formula between the open circuit potential (*E*_ocp_) and the zero charge potential (*E*_pzc_). It has been proved that the potential of zero charge (PZC) is a significant electrochemical property of the metal and plays a significant role in the electrical bilayer structure in electrocapillary and electrokinetic phenomena, wetting phenomena, adsorption of ions and neutral organic molecules on the electrode, and physicochemical mechanics.^[^ ^45^ ^]^ The metal's net surface charge is found with the following equation

(1) $$Er=E_{ocp}-E_{pzc}$$

here, *E*r represents Antropov's rational potential for corrosion.^[^ ^46^ ^]^ If *E*r is negative because of the equation mentioned above, the electrode surface becomes negatively charged and adsorption of cations takes place. If *E*r is positive, the electrode surface becomes positively charged and adsorption of anions takes place. The peak of the curve obtained from 0.5 M HCl solution containing 80 ppm quinic acid (**Figure** **15**) is the zero charge potential (PZC) and its value is −0.493 V. (Ag/AgCI). Under the same conditions, the OCP is −0.483 V (Ag/AgCI). In this case, *E*r (Ag/AgCI) =  +0.01 V. It shows that the surface of the electrode is positive under the conditions studied.^[^ ^47^ ^]^ According to the PZC results, the surface charge was positive in the presence of quinic acid. Thus, it can be said that quinic acid must be in the protonated form in HCl.^[^ ^42^ ^]^

In theoretical calculations, the bandgap was found as −5.986 eV, *E*_LUMO_ −3.337 eV; *E*_HOMO_: −9.324 eV (Table 1). Bandgap (Δ*E*: 5.986 eV) is an indicator of the activity of quinic acid as a corrosion inhibitor. As this value decreases, activity and electron donation increase.^[^ ^48^ ^]^ A higher *E*_HOMO_ value (−9.324) (less negative) or a lower *E*_LUMO_ value (highly negative) indicates that the adsorption process can take place efficiently on the MS surface.^[^ ^48^ ^]^ Low dipole moment values allow molecules to gather on the MS surface and adsorption to take place more.^[^ ^48^ ^,^ ^49^ ^]^ µ: −0.039 value for quinic acid may give information about the adsorption of this compound. Softness was 1.496 eV and hardness 2.993 eV. Lower Δ*E* makes the compound less hard, so it can be said that the softer compound in terms of hardness and softness tends to give electrons to the MS surface and has a high adsorption ability. Lower electronegativity is related to higher electron transfer. High electron transfer indicates that high adsorption to the MS surface occurs. Consequently, it is thought to be effective in helping good inhibition and corrosion protection.^[^ ^48^ ^,^ ^50^ ^,^ ^51^ ^]^

According to EIS parameters, the effectiveness of quinic acid's inhibition increased with increasing concentration. MS showed high resistance due to the inhibitory effect of quinic acid at low concentrations. This result is evidence for the corrosion inhibition of quinic acid. Quinic acid can effectively protect MS in HCl solution against corrosion during long‐term immersion. The protective layer formed due to H bonds between OH groups in the molecular structure of quinic acid and H_2_O and HCl molecules is probably effective in this result. The calculated quantum parameters revealed that quinic acid effectively inhibits corrosion due to its low bandgap and larger dipole moment values.

## 3 Conclusions

EIS parameters showed that the inhibition efficiency of quinic acid increased proportionally with increasing concentration. During long‐term submergence, quinic acid, which was determined to form a hydrophilic coating on the MS, can effectively protect the MS against corrosion caused by the HCl solution. In the mixture with the inhibitor, the net surface charge (Er) of the metal is positive, indicating that the electrode surface is positive under these conditions. All results showed that quinic acid has a corrosion inhibitory effect at an increasing rate with increasing concentration. In the field of corrosion studies, quinic acid has been found to have potential applications in the field of MS protection and stabilization, especially in HCl media in the long term.

## 4 Experimental Section

4.1

4.1.1

4.1.1.1

D‐quinic acid and HCl were acquired from Sigma Aldrich and Merck KGaA, respectively, for use in the research. First, molecular orbital calculations (DFT and ESP) analyses of quinic acid were performed. Then, quinic acid solutions were prepared with distilled water at 10, 20, 40, and 80 ppm concentrations. For a set amount of time (1 or 144 h), a MS electrode was submerged in these solutions. Electrochemical corrosion inhibition measurements were made at different temperatures (25, 35, 45, and 55 °C). Electrochemical measurements were made using polarization curves and EIS. All electrochemical measurements were performed utilizing a Reference 3000 Gamry Potentiostat instrument. In addition, after this dipping process, the absorbance of 0.5 M HCI solution and quinic acid solutions prepared with acid were measured with SHMADZU brand UV‐3600 model Uultraviolet–visible light spectrophotometer (UV–VIS–NIR). The effect of quinic acid on SEM images and EDX analyses were examined using JEOL JSM 6510 brand model SEM equipment and Park System XE‐100E AFM to assess the surface morphology of MS. Using a standard three‐electrode setup, electrochemical measurements were made. The working electrode was constructed from MS (carbon steel). In terms of weight percentage, the MS electrode's chemical makeup was as follows: 0.057 C, 0.038 P, 0.320 Si, 0.335 S, 1.900Mn, 17.900 Cr, 0.170 Cu, 8.150 Ni, 0.090 Mo, and 71.040 Fe. The exposed area of the MS electrode was 0.785 cm^2^. As a counter electrode (with a surface area of 2 cm^2^) and reference electrode, respectively, a platinum sheet and an Ag/AgCl (3 M KCl) electrode were utilized. The MS electrode was ground mechanically with 1200 grit abrasive paper before each experiment, and it was then cleaned by being submerged in a 1:1 ethanol: acetone mixture after rinsing in distilled water. The Gamry Potentiostat electrochemical analyzer was used to carry out the electrochemical tests. Quinic acid's contact angle with MS was also computed to ascertain whether the surface was hydrophobic or hydrophilic via Biolin Scientific, Theta Lite.^[^ ^44^ ^,^ ^52^ ^]^

##### Statistical Analysis

The one‐way ANOVA test used in the triplicate analyses revealed that quinic acid solutions significantly reduced the corrosion of mild steel (*p* < 0.05).

## Conflict of Interest

The authors declare no conflict of interest.

## Author Contributions

**Serap Toprak Döşlü**: conceptualization (equal); formal analysis (equal); investigation (equal); methodology (equal). **Leyla Ercan**: data curation (equal); formal analysis (equal); software (lead); supervision (equal).

## Data Availability

The data that support the findings of this study are available from the corresponding author upon reasonable request.
